# Inhibition of SREBP increases gefitinib sensitivity in non-small cell lung cancer cells

**DOI:** 10.18632/oncotarget.10721

**Published:** 2016-07-20

**Authors:** Jiajin Li, Hui Yan, Li Zhao, Wenzhi Jia, Hao Yang, Liu Liu, Xiang Zhou, Ping Miao, Xiaoguang Sun, Shaoli Song, Xiaoping Zhao, Jianjun Liu, Gang Huang

**Affiliations:** ^1^ Department of Nuclear Medicine, Ren Ji Hospital, School of Medicine, Shanghai Jiao Tong University, Shanghai 200127, China; ^2^ Institute of Clinical Nuclear Medicine, Ren Ji Hospital, School of Medicine, Shanghai Jiao Tong University, Shanghai 200127, China; ^3^ Institute of Health Sciences, Shanghai Jiao Tong University School of Medicine & Shanghai Institutes for Biological Sciences, Chinese Academy of Sciences, Shanghai 200025, China; ^4^ Shanghai University of Medicine and Health Sciences, Shanghai 201318, China

**Keywords:** lung cancer, gefitinib, SREBP, chemotherapy, lipid metabolism

## Abstract

The clinical success of EGFR inhibitors in patients with lung cancer is limited by the inevitable development of treatment resistance. Here, we show that inhibition of SREBP increase gefitinib sensitivity *in vitro* and *in vivo*. Interference of SREBP1 binding partner MARVELD1 potentiate the therapeutic effect of gefitinib as well. Mechanistically, SREBP inhibition decreases the cell membrane fluidity, results in a decreased tyrosine phosphorylation of EGFR. Therefore, targeting lipid metabolism combined with EGFR-TKIs is potentially a novel therapeutic strategies for cancer treatment.

## INTRODUCTION

Lung cancer is world widely the leading cause of cancer related death [1]. Non-small cell lung cancer (NSCLC) accounts for the majority (85%) of all cases. Epidermal growth factor receptor (EGFR) tyrosine kinase inhibitors (TKIs), such as gefitinib, are successful treatments for the approximately 10% of NSCLC with an activating EGFR mutation, but have limited efficacy in an EGFR wild-type unselected population. The use of novel combination regimens to avoid resistance might pave the way to achieve durable benefits for majority of the patients.

Cancer cells often have characteristic changes in metabolism. We previously demonstrated targeting cellular metabolism is a potent therapeutic strategy to overcome drug resistance in lung cancer [2, 3]. Cellular proliferation, a common feature of all cancers, requires fatty acids for synthesis of membranes and signaling molecules. To date, several inhibitors targeting the fatty acid biosynthesis pathway have shown antitumor activity [4]. The promising results with metabolic inhibitors provide another avenue to improve the efficacy of EGFR TKIs in NSCLC [5].

Sterol regulatory element binding proteins (SREBPs) are a family of transcription factors that activate lipid homeostasis by controlling the expression of a range of enzymes required for endogenous cholesterol, fatty acid, triacylglycerol and phospholipid synthesis. There are three forms of SREBP in mammals: SREBP-1a, -1c and -2. Although they undergo similar proteolytic activation and share some target genes, SREBP-1c mainly stimulate fatty acid synthesis, whereas SREBP-2 is relatively specific to cholesterol synthesis. The SREBP-1a isoform is implicated in both pathways [6].

In this study, we present that inhibition of SREBP pathway with small molecular inhibitors, Betulin, fatostatin and 25-hydroxycholesterol (25HC) respectively, enhances the gefitinib sensitivity of NSCLC cells. Same result have confirmed by siRNA. Furthermore, we identify MARVELD1 as a SREBP binding partner. Interference of MARVELD1 inhibit SREBP depended lipogenesis and improve the efficacy of gefitinib in NSCLC. Those results highlight that targeting SREBP combined with EGFR TKIs is potentially an effective therapeutic strategy.

## RESULTS

### SREBP inhibition enhance gefitinib sensitivity in lung cancer cells

**Figure 1:**
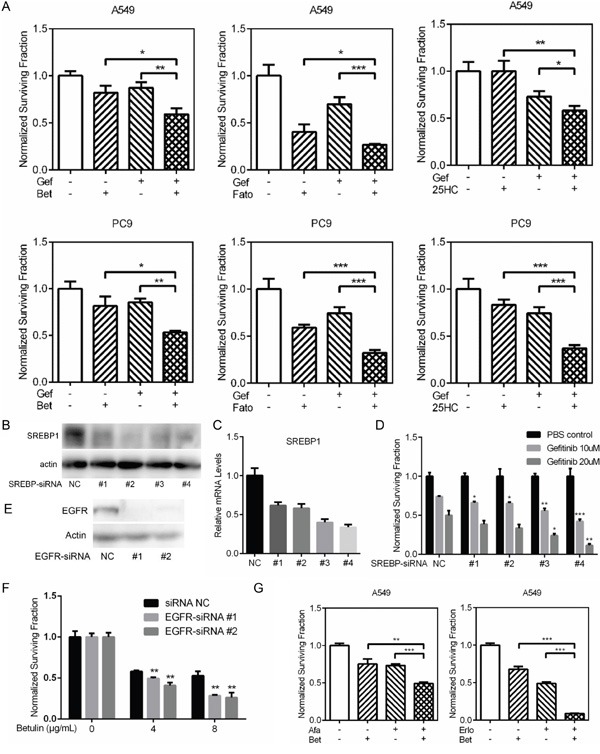
The effects of SREBP inhibition on gefitinib sensitivity **A.** A549 and PC9 cells were treated with indicated SREBP inhibitor (betulin 4 ug/mL, fatostatin 4 ug/mL and 25-HC 4 ug/mL) alone or combined with gefitinib (A549 10μM and PC9 5μM) for 48h. Relative cell viability was measured and calculated as described in “Materials and Methods”. Treatments by inhibitor combined with gefitinib have greater antiproliferative effect than each drug alone in both A549 and PC9 cells. **B.** A549 cells were transfected with NC-siRNA or SREBP1-siRNA. Two days after transfection, the protein levels of SREBP1 were examined by western blot, **C.** The mRNA levels of SREBP1 were examined by quantitative RT-PCR (C). **D.** The transfected A549 cells were treated with indicated concentration of gefitinib for 48h. Relative cell viability was measured and calculated as above. **E.** A549 cells were transfected with NC-siRNA or EGFR-siRNA. Two days after transfection, the protein levels of SREBP1 were examined by western blot. **F.** The transfected A549 cells were treated with indicated concentration of betulin for 48h. Relative cell viability was measured and calculated as above. **G.** A549 cells were treated with indicated EGFR TKI (afatinib 5μM, erlotinib 20μM) alone or combined with betulin (4 ug/mL). Relative cell viability was measured and calculated as above. Data are means ± SD (n = 3). *P < 0.05, **P < 0.01, ***P < 0.005 by t-test.

Betulin, fatostain and 25-hydroxycholesterol were previously identified as inhibitors of SREBP pathway [7–9]. To examine whether SREBP inhibitors has synergism or additive effects with gefitinib, we combined each drug with suboptimal doses of gefitinib. Combination of SREBP inhibitors with a suboptimal dose of gefitinib significantly potentiates the cytotoxic effect of gefitnib in A549 and PC9 (Figure 1A).

To further confirm that the potentiating effect of SREBP inhibition on gefitinib, we knockdowned SREBP1 using four independent siRNAs (Figure 1B). Silencing of SREBP1 resulted an enhanced cell growth inhibition after gefitinib treatment, and the potentiating effect is related to SREBP1 knockdown efficiency (Figure 1C and 1D). To further confirm that the synergistic effects are between EGFR signaling and the SREBP pathway, two more EGFR inhibitors, afatinib and erlotinib, were combined with betulin to treat A549. As shown in Figure 1G, combination treatments resulted in strong anti-proliferative effects. Moreover, A549 transfected with EGFR siRNAs were validated to be more sensitive to betulin than negative control (Figure 1E and 1F).

**Figure 2:**
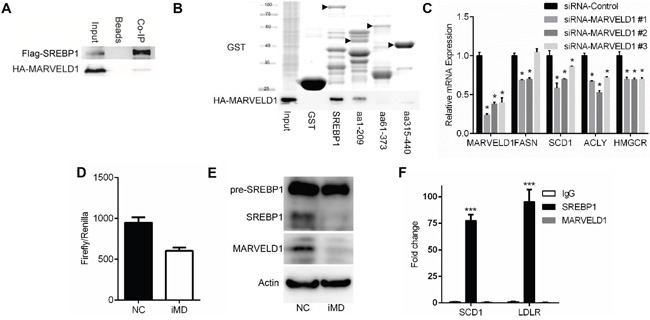
MARVELD1 interacts with SREBP1 **A.** HEK293T cells were co-transfected with Flag-SREBP1a and HA-MARVELD1. After 48 h of incubation, cell lysates were prepared in IP buffer and Flag-tagged proteins were immunoprecipitated with anti-Flag antibody (IgG as control). The presence of Flag- or HA-tagged proteins were analyzed by immunoblotting using anti-HA or anti-Flag antibody. **B.** HEK293T cell were transfected with HA-MARVELD1. After 48h, cell lysates were incubated with GST-fusion proteins of the indicated fragments of SREBP1a in GST pulldown assays. GST alone was a negative control. Bound HA-MARVEDL1 proteins were analyzed by immunoblotting using anti-HA antibody. **C.** Effects of MARVELD1 interference (or siRNA-NC as the control) on the SREBP target gene expression in A549 cells were analyzed by q-PCR. **D.** Effects of MARVELD1 interference on the SREBP transcriptional activity in A549 cells were analyzed using the Dual Luciferase assay system. **E.** Effects of MARVELD1 interference on the SREBP protein level. **F.** Chromatin immunoprecipitation (ChIP) assay was performed using antibodies against SREBP-1, MARVELD1 and IgG. Sequences were amplified by qPCR.

MARVELD1 is previously reported as a drug-resistant related protein [10]. Here, we validated the interaction between SREBP and MARVELD1 by co-immunoprecipitation (Figure 2A). Pull-down showed MARVELD1 only bound to the GST-fusion proteins containing aa 1–60 of SREBP1, which is the transcriptional activity domain of the SREBP1 (Figure 2B). qRT-PCR analyses revealed that MARVELD1 knockdown significantly downregulated the mRNA levels of classical SREBP-1a target genes, including *Fasn*, *Scd1*, *Acly* and *Hmgcr* (Figure 2C). siRNA-MARVELD1 #1 led to an 76% reduction in *Marveld1* expression and was selected as the most efficient siRNA for use in this study. As shown in Figure 2D, MARVELD1 knockdown significantly decreased the SREBP1 activity as analyzed by luciferase reporter assays. Western blotting showed the mature form of SREBP1 was significantly decreased after MARVELD1 interference (Figure 2E). However, results of chromatin immunoprecipitation (ChIP) suggested that MARVELD1 is not directly present on SREBP1 target promoters (Figure 2F). These results demonstrated that MARVELD1 knockdown decrease the SREBP1 protein level and thus inhibit transcription activity.

**Figure 3:**
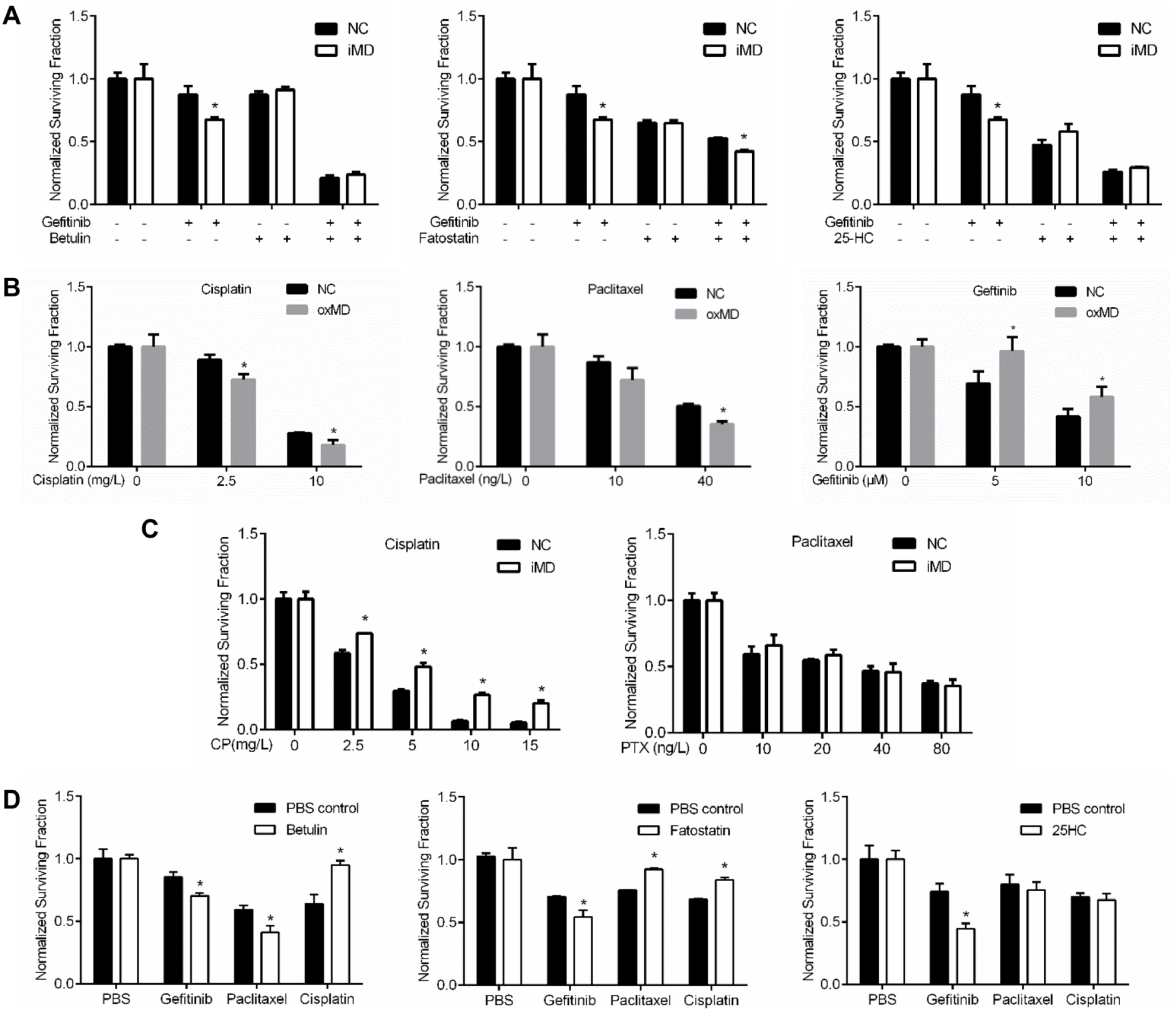
MARVELD1 modulate gefitinib sensitivity **A.** A549 cells transfected with NC-siRNA or MARVELD1-siRNA were incubated with gefitinib in the presence of indicated SREBP inhibitor. Relative cell viability was measured after 48h. Interference of MARVELD1 enhances gefitinib sensitivity, and the potentiating effects were attenuated in the presence of SREBP inhibitor. **B.** Effects of overexpressing MARVELD1 (or empty vector as the control) on the sensitivity of cisplatin, paclitaxel or gefitinib in A549 cells were analyzed as above. **C.** Effects of MARVELD1 interference (or siRNA-NC as the control) on the sensitivity of cisplatin, paclitaxel or gefitinib in A549 cells were analyzed as above. **D.** Effects of SREBP inhibitors incubation (or PBS as the control) on the sensitivity of gefitinib, paclitaxel and cisplatin in A549 cells were analyzed as above. Data are means ± SD (n = 3). *P < 0.05.

Next, we asked if MARVELD1 knockdown has similar effect as SREBP inhibitors on gefitinib sensitivity. As shown in Figure 3A, A549 cells transfected with MARVELD1 siRNA were more sensitive to genfitinib than negative control. The potentiation effect were attenuated when the cells were co-treated with SREBP inhibitors, which suggesting that the modulation effect of MARVELD1 on gefitinib sensitivity was depended on SREBP.

However, it has been reported that overexpression of MARVELD1 promotes the tumor sensitivity to chemotherapy in hepatocellular carcinoma, and our results seems conflicted with previous study [10]. To clarify this, we assessed the sensitivity of A549 cells to a serial chemo-drugs by overexpression of MARVELD1. As shown in Figure 3B, similar with the previous study, cisplatin and paclitaxel sensitivity were enhanced by marveld1 overexpression, but on the contrary, gefitinib sensitivity was decreased. To further testify this, we apply siRNA to inhibit MARVELD1. As shown in Figure 3C, A549 cells became resistant to cisplatin in MARVELD1 knockdown group, and the paclitaxel sensitivity was not significantly changed. These results suggest that the effects of MARVELD1 on different chemo-drugs are not the same. Inhibition of MARVELD1 antagonize cisplatin and paclitaxel resistance but potentiate geftinib.

We next asked if SREBP inhibitors also have different effects on the sensitivity of those chemo-drugs. As shown in Figure 3D, gefitinib was potentiated by all the three inhibitors. However, betulin potentiated paclitaxel but antagonized cisplatin, fatostatin antagonized both paclitaxel and cisplatin, and 25-HC had no effect on the sensitivity of the two drugs. These results suggested that the effects of those three inhibitors on paclitaxel and cisplatin are not specific and may not by inhibition of SREBP, thus we focus our study on gefitinib.

### Effects of SREBP inhibitors on lipid metabolism and SREBP transcriptional activity in NSCLC cells

**Figure 4:**
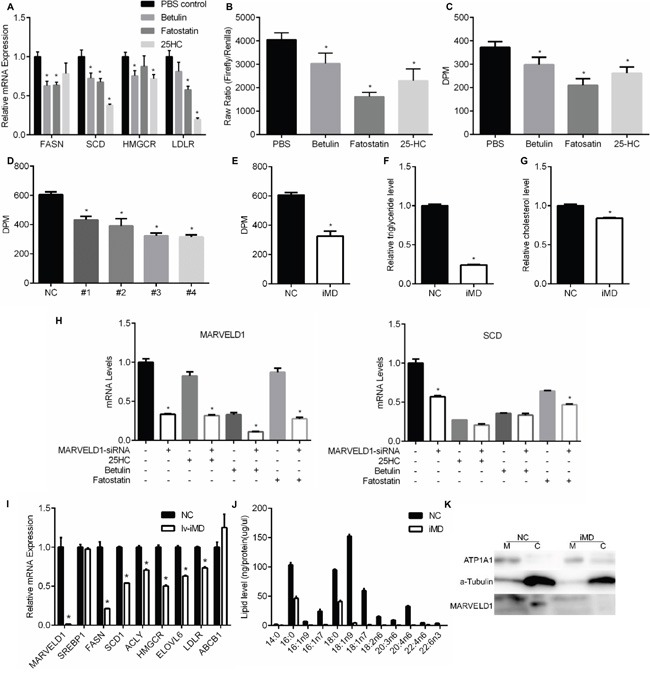
Effect of SREBP inhibition on lipid metabolism **A.** qRT-PCR was used to analyze the effects of SREBP inhibiors on the mRNA levels of indicated genes in A549 cells. **B.** Dual Luciferase assays were used to examine the effects of SREBP inhibitors on SREBP transcription activity in A549 cells. **C.** Effects of SREBP inhibitors on de novo lipogenesis. Incorporation of [U-^14^C] glucose into the lipid fraction was measured in the A549 cells treated with SREBP inhibitors. **D.** Incorporation of [U-^14^C] glucose into the lipid fraction was measured in the A549 cells transfected with SREBP-siRNAs or siRNA-NC as control. **E.** Incorporation of [U-^14^C] glucose into the lipid fraction was measured in the A549 cells transfected with MARVELD1-siRNAs or siRNA-NC as control. **F.** Influence of MARVELD1 knockdown on intracellular triglyceride level. **G.** Influence of MARVELD1 knockdown on intracellular cholesterol level. **H.** qRT-PCR was used to analyze the effects of siRNA-MARVELD1 (or siRNA-NC as the control) on the mRNA levels of indicated genes in the presence of SREBP inhibitors (or PBS as the control) in A549 cells. **I.** Interference of MARVELD1 by lentivirus markedly reduced the SREBP target genes mRNA levels but had no influence on SREBP and non-SREBP-target gene ABCB1. **J.** Membrane lipid metabolites of A549 cells in (I) were analyzed by gas chromatography-mass spectrometer (GC-MS) and normalized by total protein levels. **K.** Western blot of membrane and cytosolic fractions of A549 cells in (J). MARVELD1 is detectable in cytosolic (C) fractions of NC group. ATP1A1 was used as the membrane marker, a-Tubulin as cytosolic marker to exclude contamination during cell fraction isolation. Data are means ± SD (n = 3). *P < 0.05.

Compared with cisplatin and paclitaxel, gefitinib as an EGFR TKI has more tightly connections with lipid metabolism as revealed in previous studies [5]. To verify the small molecular drugs we used could effectively inhibit SREBP pathway, we examined several SREBP targeted gene expression after drug treatment. As shown in Figure 4A, all the four genes we examined involved in lipid metabolism, *Fasn*, *Scd*, *Hmgcr* and *Ldlr*, were significantly down-regulated by betulin, fatostatin and 25-hydroxycholesterol. The SREBP transcription activity was also down-regulated as confirmed by luciferase assay (Figure 4B). To measure specific effects on de novo lipid synthesis, cells were labeled with 14C-glucose. Consistently, all the three drugs induced a decrease in the incorporation of glucose into lipid (Figure 4C). In Figure 4D, we showed that the SREBP1 siRNA we used effectively inhibit lipogenesis in A549 cells.

To determine whether knockdown of MARVELD1 has a crucial role on lipid accumulation, A549 cells transfected with MARVELD1 siRNA or negative siRNA were cultured in DMEM media for 24h. As depicted in Figure 4F and 4G, the siRNA knockdown of MARVELD1 resulted in a significant 60% to 70% decrease in cellular triglyceride, and 10% to 20% decrease in cholesterol. Figure 4E showed a 40% to 50% decrease in de novo lipogenesis with MARVELD1 siRNAs as compared with negative siRNA. To test if MARVELD1 siRNA induced down-regulation of SREBP target gene mRNA level is dependent on SREBP pathway. We examined the expression of SREBP target gene *Scd* after MARVELD1 siRNA transfection in the presence of SREBP inhibitors. As shown in Figure 4H, the inhibiting effect of MARVELD1 siRNA on *Scd* expression were attenuated when incubated with SREBP inhibitors.

Knockdown of MARVELD1 by siRNA has shown that several SREBP target gene were down regulated, such as Fasn, Scd1, Hmgcr and Ldlr (Figure 2C). However, the knockdown efficiency of *Marveld1* by siRNAs were only about 70% to 80%. To increase the efficiency of MARVELD1 knockdown, we packed marveld1 shRNA into lentivirus. As shown in Figure 4I, the *Marveld1* mRNA expression was almost completely inhibited by lentivirus, and all the SREBP targeted genes we tested were downregulated. Moreover, we found that the saturated to unsaturated fatty acids ratio were changed on the membrane in MARVELD1 inhibited cells (Figure 4J and 4K). Though all the fatty acids were decreased in MARVELD1 knockdown cell membrane, unsaturated fatty acids decreased more significant compared with saturated.

### SREBP inhibition decrease cell membrane fluidity and EGFR signaling in NSCLC cells which is rescued by oleic acid

**Figure 5:**
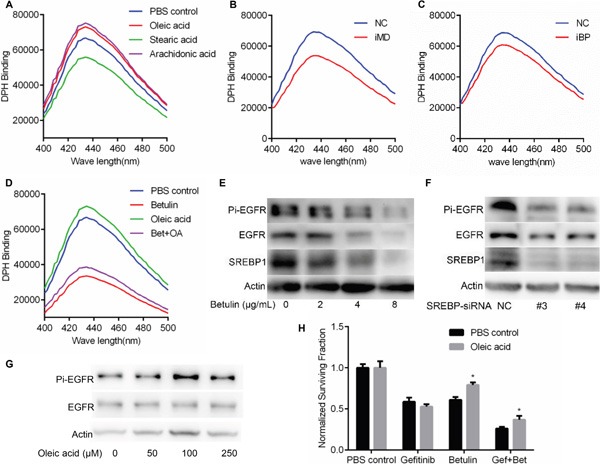
Effect of lipogenesis inhibition on cell membrane fluidity and EGFR phosphorylating activity **A.** The fluorescence excitation spectrum of DPH was examined in A549 cells treated with indicated fatty acid. **B.** Effect of MRVELD1 interference on the amounts of DPH bound to A549 cells. **C.** Effect of SREBP interference on the amounts of DPH bound to A549 cells. **D.** Betulin incubation markedly reduced the amounts of DPH bound to A549 cells and was partially rescued by oleic acid. **E.** A549 cells were incubated with for indicated concentration of betulin, cell lysates were prepared and analyzed by immunoblotting using the indicated antibodies. **F.** A549 cells were transfected with siRNA-SREBP (or siRNA-NC as the control). Samples were analyzed 48 h after transfection and the levels of actin were used as the loading control for immunoblotting. **G.** A549 cells were treated with indicated concentration of oleic acid. After 4h of incubation, cell lysates were prepared and analyzed by immunoblotting using the indicated antibodies. **H.** Effect of betulin on gefitinib sensitivity with or without oleic acid was examined as above. Data are means ± SD (n = 3). *P < 0.05.

Unsaturated fatty acids have been reckoned as membrane fluidizer [11]. It was reported that the kinase activity of reconstituted EGFR was decreased by lower membrane fluidity [12]. Along this line, we asked if the membrane fluidity were decreased by MARVELD1 or SREBP inhibition thus suppressing the EGFR signaling. To evaluate the cell membrane fluidity, the amounts of DPH bound to 10^6^ cells incubated with 16μM DPH at 25°C for 30 min were compared. It was found that betulin treated A549 cells bind 50% to 60% less DPH than non-treatment group, and was partially rescued by oleic acids (Figure 5D). Consistently, interference of both MARVELD1 (Figure 5B) and SREBP1 (Figure 5C) reduced the amounts of DPH bound to cell membrane. These results support the conclusion that inhibition of SREBP decreases the fluidity of cell membrane in NSCLC.

Membrane fluidizing effect of fatty acids was dependent on chain length and unsaturation. Short chain fatty acids were inactive, and PUFAs were more effective (Figure 5A). In the present study, we used preferentially OA (although less potent than PUFA) because OA is the major cellular UFA. To test if the EGFR tyrosine kinase activity was modulated by SREBP inhibition, A549 cells were infected with SREBP1 siRNA or negative control siRNA. Silencing of SREBP1 was associated with decreases in phosphorylation of EGFR (Figure 5F). Consistently, betulin suppressed the phosphorylation of EGFR as well (Figure 5E). Furthermore, EGFR phosphorylation increased progressively with OA concentration, apparently without saturation up to 100 mmol/L (Figure 5G).

To investigate whether betulin enhanced gefitinib sensitivity could rescued by oleic acids, A549 cells were incubated with gefitinib, betulin or both in the presence of oleic acids or PBS as control, after 48h cell viability was then detected. As shown in Figure 5H, oleic acids did not change the gefitinib sensitivity of A549 cells, but attenuated betulin induced cell growth inhibition. Importantly, the potentiation effect of betulin on gefitinib was thus attenuated.

### Betulin potentiate gefitnib *in vivo*

**Figure 6:**
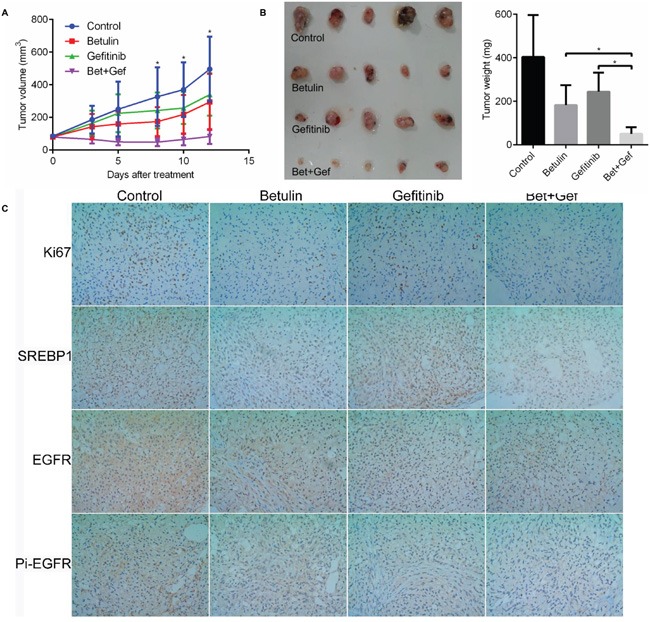
SREBP inhibitor betulin potentiate gefitinib *in vivo* Nude mice were injected with 5 × 10^6^ A549 cells and treated with control, betulin, gefitinib or betulin combined with gefitinib. **A.** The growth curve of tumors *in vivo*; **B.** image of tumors isolated from those mice and the weight of tumors when mice were killed; **C.** representative images of Ki-67, SREBP1, EGFR and pi-EGFR staining of tumor samples. *P<0.05.

To extend the find that SREBP inhibition increase gefitinib sensitivity, here we sought to determine whether concurrent betulin exposure could potentiate gefitinib *in vivo*. Initially, nude mice bearing A549 xenografts received control, betulin, gefitinib or betulin and gefitinib combination therapy. Although tumor growth was reduced in mice treated with each agent alone, tumors still increased in size by 3-fold (Figure 6A). In striking contrast, growth of the tumors in animals treated with the combination was completely arrested, and the size of the tumors was significantly smaller than other groups (Figure 6B). All treatments were well tolerated, with no significant loss of body weights observed over the 2 weeks of dosing. These data demonstrate that combining the SREBP inhibitor betulin with gefitinib dramatically reduces tumor growth *in vivo*.

Immunohistochemical analysis of the four groups of A549 xenograft tumors was conducted to testify if the SREBP or EGFR were effectively inhibited by their targeting drugs. As shown in Figure 5C, in groups treated with betulin or betulin+gefitinib, the SREBP1 expression level were significantly lower than control. Similarly, tumors treated with gefitinib or betulin+gefitinib had lower expression in EGFR and phosphorylated EGFR. Besides, betulin treatment also induced EGFR inhibition, which is consistent with our previous data *in vitro*. These data demonstrate that in the doses we applied to treat the mice, those drugs inhibited their according targets effectively.

Our previous data showed that betulin augments the ability of gefitinib to reduce cell proliferation. In the tumors studied here, there was a small decrease in Ki67 staining from tumors of mice treated with betulin or gefitinib monotherapy. In contrast, tumors from mice treated with the betulin and gefitinib combination showed a dramatic reduction in Ki67 (Figure 5C). This further confirm that tumors treated with both betulin and gefitinib were less proliferative than single agent treatment. These results indicated that gefitinib plus betulin exerts a substantial chemotherapeutic effect to potentiate gefitinib effect on xenograft growth in mice.

## DISCUSSION

Gefitinib, an epidermal growth factor receptor targeting drug, has been clinically useful for the treatment of patients with NSCLC. However, success rates vary between different tumor types, and thus it is important to understand which molecular target(s) are responsible for limiting the therapeutic efficacy of the drug [13].

Metabolic reprogramming is a hallmark of cancer [14]. Targeting metabolism could improve existing approaches [15]. In this study, our data show that betulin, fatostain and 25-hydroxycholesterol effectively inhibit de novo lipid synthesis and potentiate gefitinib in NSCLC cells. These three small molecular drugs share a common inhibiting target SREBP.

Sterol regulatory element binding proteins (SREBPs) are a family of transcription factors that regulate lipid homeostasis by controlling the expression of a range of enzymes required for endogenous cholesterol, fatty acid (FA), triacylglycerol and phospholipid synthesis [6]. SREBP1 has an important role in cancer progression likely by providing the membrane building materials to support the rapid proliferation of cancer cells [16, 17]. We have utilized several experimental approaches to identify novel SREBP interacting proteins [18, 19]. Here, we presented a novel interaction between the nuclear protein MARVELD1 and the nuclear form of SREBP1. Our biochemical analyses demonstrate MARVELD1 directly binds to transcription activity domain of SREBP1. Multitude of evidence show that interference MARVELD1 binding to SREBP1 decreases the expression of lipogenic genes including *Scd1*, and subsequent accumulation of lipids in A549 cells. Moreover, MARVELD1 knockdown resulted in an enhanced gefitinib sensitivity which is SREBP1-dependent.

Membrane-bound receptors are activated by external molecules and transmit signals across the membrane. Changes in membrane lipid composition, particularly its MUFA content, are involved in cellular responses and in modulating intracellular signaling [20]. As a plasma membrane-resident protein, EGFR activation and function is modulated by its surrounding lipid micro-environment [21]. It has been reported that migration of EGFR to more fluid compartments may be required for its functional activation [22]. Moreover, exogenous polyunsaturated fatty acids were shown to alter the partition of EGFR in membrane and increase the phosphorylation of the receptor [23]. Membrane fluidity depends on the level of unsaturation of membrane lipids [11]. Our results show that inhibition of SREBP accompanied with decrease in EGFR phosphorylating activity and cell membrane fluidity. Furthermore, oleic acid incubation rescue the decrease of SREBP inhibition induced membrane fluidity, and results in more active EGFR phosphorylation. Thus attenuate the potentiating effects of SREBP inhibitors on gefitinib sensitivity in lung cancer cells.

In summary, our data demonstrated a theoretical model of SREBP inhibition enhance therapeutic response of lung cancer cells to EGFR-TKI gefitinib. SREBP activates the expression of lipogenic genes, thus modulates membrane lipid composition and sustains membrane unsaturated fatty acids proportion. Reduced proportion of unsaturated fatty acids on the membrane results in a decreased cell membrane fluidity, thereby inhibit the activity of membrane-bound receptor EGFR and synergize with gefitinib. Furthermore, we showed that MARVELD1 binds to SREBP1 and stimulates lipid metabolism. Interference of MARVELD1 enhances the sensitivity of lung cancer cells to gefitinib as well. Taken together, our study has implied that inhibition of SREBP-driven lipogenic program combined with EGFR TKIs is a promising therapeutic strategy for non-small cell lung cancer.

## MATERIALS AND METHODS

### Materials

Cell culture reagents (DMEM and fetal bovine serum) were from Invitrogen/Gibco. [U-14C]-glucose were from Shenzhen Zhonghe Headway Bio-Sci & Tech Co. Anti-Flag M2 (Sigma- Aldrich, St Louis, MO, USA), monoclonal anti-HA (Covance, Dedham, MA, USA), anti-SREBP-1 (Santa Cruz Biotechnology, Santa Cruz, CA, USA), anti-β-actin (Cell Signaling Technology, Danvers, MA, USA) anti-EGFR (Cell Signaling Technology) and anti–pi-EGFR (Cell Signaling Technology) antibodies were purchased. Oleic acid, stearic acid and arachidonic acid were purchased from Sigma-Aldrich. Lipofectamine 2000 (Invitrogen, Carlsbad, CA, USA) was used in transient transfection according to manufacturer's protocol. Paclitaxel (PTX) was from Bristol-Myers Squibb. Cisplatin and gefitinib were obtained from Selleck Chemicals.

### Cell culture

The human NSCLC cell line PC9 and A549 (Cell Bank of the Chinese Academy of Sciences, Shanghai, China) were maintained in DMEM medium. The media were supplemented with 10% FBS and 100 units/mL penicillin/streptomycin. Cell cultures were maintained in 5% CO_2_ and air in a humidified 37°C incubator. Cells plated in plastic culture dishes were treated with drugs 1 day after plating, and the drugs were present throughout the indicated incubation periods.

### Measurement of cell viability

Cells were seeded in 96-well plates at 5×10^3^ cells/well. After 24h, cells were treated with various drugs and incubate for 48h. Cell viability was determined using the CCK-8 assay (Dojindo) according to the manufacturer's instructions, and normalized to the non-treatment control group.

### Transfection of siRNA

Cells were transfected with oligo siRNAs using Lipofectamine 2000. The sequences of siRNA oligos used in this study are as follows:

**Table:** 

siRNA	Sense (5′-3′)	Antisense (5′-3′)
Non-specific	UUCUCCGAACGUGUCACGUTT	ACGUGACACGUUCGGAGAATT
SREBP1 #1	GCGCACUGCUGUCCACAAATT	UUUGUGGACAGCAGUGCGCTT
SREBP1 #2	GCACUGAGGCAAAGCUGAATT	UUCAGCUUUGCCUCAGUGCTT
SREBP1 #3	GCUGAAUAAAUCUGCUGUCUUTT	AAGACAGCAGAUUUAUUCAGCTT
SREBP1 #4	GCUGCAUUGAGAGUGAAGATT	UCUUCACUCUCAAUGCAGCTT
MARVELD1 #1	CCUGCUUUCGGCGCUCUAUTT	AUAGAGCGCCGAAAGCAGGTT
MARVELD1 #2	CCAGCCUCCUUAAUCCCUUTT	AAGGGAUUAAGGAGGCUGGTT
MARVELD1 #3	GUUGUACCCUUACACUUGUTT	ACAAGUGUAAGGGUACAACTT
EGFR #1	GAUCUUUCCUUCUUAAAGATT	UCUUUAAGAAGGAAAGAUCAT
EGFR #2	GGAAAUAUGUACUACGAAATT	UUUCGUAGUACAUAUUUCCTT

### mRNA expression analysis

Total RNA was isolated using a Trizol kit (Omega, Norcross, GA, USA) and transcribed to cDNA with a cDNA synthesis kit (Takara, Otsu, Japan). Quantitative real-time PCR was performed using SYBR Green PCR Master Mix (Takara) and the transcript levels of genes were detected by using the StepOnePlus Real-Time PCR System (Applied Biosystems, Foster City, CA, USA). Primers used for detection of specific genes are listed below.

**Table:** 

Gene name	Forward primer (5′-3′)	Reverse primer (5′-3′)
MARVELD1	TCACTATCGCCACCAGCAAG	GCAGCGTGAGGAAGTAGAGG
SREBP1	GCTGACCGACATCGAAGACA	CTGCCTGGGGAGCTGGTATC
FASN	TATGAAGCCATCGTGGACGG	CATGCTGTAGCCCACGAGT
SCD1	CACTTGGGAGCCCTGTATGG	TGAGCTCCTGCTGTTATGCC
ACLY	CAGTCCCAAGTCCAAGATCCC	GTCTCGGGAGCAGACATAGT
HMGCR	GCCCTCAGTTCCAACTCACA	TTCAAGCTGACGTACCCCTG
ELOVL6	GCTAAGCAAAGCACCCGAAC	GGAGCACAGTGATGTGGTGA
LDLR	GGTCCACATTTGCCACAACC	ATGTTCACGCCACGTCATCC
ABCB1	TTGCTGCTTACATTCAGGTTTCA	AGCCTATCTCCTGTCGCATTA

### Immunoblotting

Cells were lysed into the RIPA buffer containing protease inhibitors by incubating on ice for 30 min followed by centrifugation at 10 000 g for 15 min. The extracted proteins were subjected to electrophoresis on SDS-polyacrylamide gels and transferred to PVDF membranes (GE Healthcare, Buckinghamshire, UK), which were blocked and probed with specific primary antibodies with appropriate dilution at 4°C overnight. The membranes were then incubated with the horseradish peroxidase-conjugated secondary antibodies for 1h at room temperature, followed by three washes with 1 × TBST, the immunoreactive bands were visualized by ECL Plus system (Tanon, Shanghai, China).

### Co-immunoprecipitation

30 μl of anti-Flag M2 affinity gel (Sigma-Aldrich) was washed as instructed, and incubated with cell extracts in IP buffer and shaking for 3 h at 4°C. The beads were washed three times with IP buffer. Eluted proteins were subjected to SDS-PAGE and detected using specific antibodies.

#### GST pulldown assay

GST-fused proteins were expressed in E. coli BL21 and purified using glutathione-sepharose 4B beads (GE healthcare, Uppsala, Sweden) according to the standard protocols. A small amount of proteins was used to verify its expression by coomassie blue staining. Cell lysates mixed with purified fused GST or GST-proteins beads at 4°C for 1 h, the beads were then washed 3 times with IP buffer. The resulting beads were analyzed by Western blots using specific antibodies.

#### Luciferase reporter assay

A plasmid containing the Sterol regulatory element (SRE) fused to the firefly luciferase gene in the pGL3 vector (Promega, Madison, WI, USA) was used. A549 or HEK293T cells with a density of 1 × 10^4^ per well were cultured in 24-well tissue culture plates and co-transfected with the firefly luciferase plasmid and a renilla luciferase plasmid (as the control) at a ratio of 10:1 in addition to overexpression plasmids or siRNA. After transfection 24–36 h, cell lysates were analyzed by the Dual Luciferase Assay system (Promega) according to the manufacturer's instructions. The ratio of firefly luciferase to renilla activity was calculated for each of the triplicates.

#### Chromatin immunoprecipitation (ChIP) assay

4 × 10^7^ cells were cross-linked with 1.42% formaldehyde for 15 minutes at RT. Formaldehyde was quenched by adding 125 mM Glycine (Sigma-Aldrich) for 5 minutes. Cells were collected, washed twice with cold PBS and lysed in IP buffer (150 mM NaCl, 50 mM Tris-HCl (pH 7.5), 5 mM EDTA, 0.5% NP-40, 1.0% Triton X-100). The crude extract was washed twice with IP buffer and sonicated 7 times for 20s. Samples were incubated overnight at 4°C with the SREBP1 (Santa Cruz), MARVELD1 (Abcam) or IgG (Cell Signalling) antibodies and then immunoprecipitated. Primers were as follows: forward SCD1 TGGAAGAGAAGCTGAGAAGG; reverse SCD1 TTCTGTAAACTCCGGCTCGT; forward LDLR GTGGGAATCAGAGCTTCACG; reverse LDLR GACCTGCTGTGTCCTAGCTG.

### *De novo* lipid synthesis

Cells grown in 6-well plates were serum starved for 16h, with 2uCi/ml 14C-glucose added to the media for the final 3h. Cells were washed twice with phosphate buffered saline before lysis in 0.5% Triton X-100. Lipids were extracted with 3:2 (v/v) hexane/isopropanol (1 ml) and quantified in duplicate samples using a LS6500 scintillation counter (Beckman Coulter, Danvers, MA, USA), and normalized to protein concentration.

### Triglyceride and cholesterol assay

Intracellular triglycerides were assayed using a triglyceride assay kit (GPO-POD; Applygen Technologies Inc., Beijing, China). Intracellular cholesterols were estimated using a cholesterol assay kit (Applygen Technologies Inc.) according to the manufacturer's recommended protocol.

### Mass spectrometry

Isolation of cell membrane was carried out using Membrane Extraction Kit according to the manufacturer's instruction (Beyotime, Haimen, China). Lipids molecules in the cell membrane lysate were tested by gas chromatography-mass spectrometer (GC-MS) (Agilent, CA, USA). The quantity of lipids was normalized to the total protein levels.

### DPH binding

From each line, 10^5^ washed cells were incubated with 16 μM DPH in PBS for 30 min at 25°C, then washed 3 times, and resuspended with PBS. DPH fluorescence was then measured using a microplate reader (Infinite M200 Pro, Tecan Group Ltd, Mannerdorf, Switzerland). The samples were excited at 365 nm, and emission measured from 400 nm to 500 nm.

### Xenograft tumor studies

All experimental procedures using animals were in accordance with the guidelines provided by the Animal Ethics Committee of Renji Hospital of Shanghai Jiao Tong University School of Medicine. A549 cells (5 × 10^6^ per injection) were subcutaneously inoculated at the right side of 4-week-old female BALB/c SCID mice (Shanghai Laboratory Animal Center, Shanghai, China). Two weeks after tumor implantation, animals were divided into two equal groups of four mice each. The first group received 100 μL gefitinib every day (50 mg/kg in 1% aqueous Tween 80) by oral gavage. The second group received gefitinib and 20 mg/kg/d of betulin. Tumor size was monitored and measured during the tumor growth for 3 weeks. Tumor volume was calculated according to the following formula: V = (length × width^2^)/2, and the results were presented as mean ± S.D. At the end of the experiments, mice were killed and tumor tissues were collected and weighed.

### Statistics

Graphpad prism software was used for statistical analysis and for plotting graphs. Results are expressed as means ±Standard Deviation (SD) of three replicate samples, and the significance of the differences between the means of treatment groups and controls was determined using Student's t-test. For statistical interpretation, P<0.05 (*) is considered significant, p<0.01 (**) is considered highly significant, and P<0.001 (***) is considered very highly significant.

## References

[1] Torre LA, Bray F, Siegel RL, Ferlay J, Lortet-Tieulent J, Jemal A (2015). Global cancer statistics, 2012. CA Cancer J Clin.

[2] Li J, Zhao S, Zhou X, Zhang T, Zhao L, Miao P, Song S, Sun X, Liu J, Zhao X, Huang G (2013). Inhibition of lipolysis by mercaptoacetate and etomoxir specifically sensitize drug-resistant lung adenocarcinoma cell to paclitaxel. PloS one.

[3] Zhou X, Chen R, Yu Z, Li R, Li J, Zhao X, Song S, Liu J, Huang G (2015). Dichloroacetate restores drug sensitivity in paclitaxel-resistant cells by inducing citric acid accumulation. Mol Cancer.

[4] Zhao Y, Butler EB, Tan M (2013). Targeting cellular metabolism to improve cancer therapeutics. Cell death & disease.

[5] Fiala O, Pesek M, Finek J, Minarik M, Benesova L, Bortlicek Z, Topolcan O (2015). Statins augment efficacy of EGFR-TKIs in patients with advanced-stage non-small cell lung cancer harbouring KRAS mutation. Tumour biology.

[6] Jeon TI, Osborne TF (2012). SREBPs: metabolic integrators in physiology and metabolism. Trends in endocrinology and metabolism: TEM.

[7] Tang JJ, Li JG, Qi W, Qiu WW, Li PS, Li BL, Song BL (2011). Inhibition of SREBP by a small molecule, betulin, improves hyperlipidemia and insulin resistance and reduces atherosclerotic plaques. Cell metabolism.

[8] Kamisuki S, Mao Q, Abu-Elheiga L, Gu Z, Kugimiya A, Kwon Y, Shinohara T, Kawazoe Y, Sato S, Asakura K, Choo HY, Sakai J, Wakil SJ, Uesugi M (2009). A small molecule that blocks fat synthesis by inhibiting the activation of SREBP. Chemistry & biology.

[9] Adams CM, Reitz J, De Brabander JK, Feramisco JD, Li L, Brown MS, Goldstein JL (2004). Cholesterol and 25-hydroxycholesterol inhibit activation of SREBPs by different mechanisms, both involving SCAP and Insigs. The Journal of biological chemistry.

[10] Yu Y, Zhang Y, Hu J, Zhang H, Wang S, Han F, Yue L, Qu Y, Zhang Y, Liang H, Nie H, Li Y (2012). MARVELD1 inhibited cell proliferation and enhance chemosensitivity via increasing expression of p53 and p16 in hepatocellular carcinoma. Cancer science.

[11] Mikami K, Murata N (2003). Membrane fluidity and the perception of environmental signals in cyanobacteria and plants. Progress in Lipid Research.

[12] Ge G, Wu J, Lin Q (2001). Effect of membrane fluidity on tyrosine kinase activity of reconstituted epidermal growth factor receptor. Biochemical and biophysical research communications.

[13] Wheeler DL, Dunn EF, Harari PM (2010). Understanding resistance to EGFR inhibitors-impact on future treatment strategies. Nature reviews Clinical oncology.

[14] Hanahan D, Weinberg RA (2011). Hallmarks of cancer: the next generation. Cell.

[15] Vander Heiden MG (2011). Targeting cancer metabolism: a therapeutic window opens. Nature reviews Drug discovery.

[16] Li W, Tai Y, Zhou J, Gu W, Bai Z, Zhou T, Zhong Z, McCue PA, Sang N, Ji JY, Kong B, Jiang J, Wang C (2012). Repression of endometrial tumor growth by targeting SREBP1 and lipogenesis. Cell Cycle.

[17] Ettinger SL, Sobel R, Whitmore TG, Akbari M, Bradley DR, Gleave ME, Nelson CC (2004). Dysregulation of sterol response element-binding proteins and downstream effectors in prostate cancer during progression to androgen independence. Cancer research.

[18] Zhu Z, Zhao X, Zhao L, Yang H, Liu L, Li J, Wu J, Yang F, Huang G, Liu J (2016). p54(nrb)/NONO regulates lipid metabolism and breast cancer growth through SREBP-1A. Oncogene.

[19] Liu L, Zhao X, Zhao L, Li J, Yang H, Zhu Z, Liu J, Huang G (2016). Arginine Methylation of SREBP1a via PRMT5 Promotes De Novo Lipogenesis and Tumor Growth. Cancer research.

[20] Vigh L, Maresca B, Harwood JL (1998). Does the membrane's physical state control the expression of heat shock and other genes?. Trends in biochemical sciences.

[21] Yuan TL, Cantley LC (2008). PI3K pathway alterations in cancer: variations on a theme. Oncogene.

[22] Pike LJ, Han X, Gross RW (2005). Epidermal growth factor receptors are localized to lipid rafts that contain a balance of inner and outer leaflet lipids: a shotgun lipidomics study. The Journal of biological chemistry.

[23] Rogers KR, Kikawa KD, Mouradian M, Hernandez K, McKinnon KM, Ahwah SM, Pardini RS (2010). Docosahexaenoic acid alters epidermal growth factor receptor-related signaling by disrupting its lipid raft association. Carcinogenesis.

